# Training general practitioners in early identification and anticipatory palliative care planning: a randomized controlled trial

**DOI:** 10.1186/s12875-015-0342-6

**Published:** 2015-09-22

**Authors:** Bregje Thoonsen, Kris Vissers, S. Verhagen, J. Prins, H. Bor, C. van Weel, M. Groot, Y. Engels

**Affiliations:** Department of Anaesthesiology, Pain and Palliative Medicine, Radboud University Medical Center, Nijmegen, The Netherlands; Department of Medical Psychology, Radboud University Medical Center, Nijmegen, The Netherlands; Department of Primary and Community Care, Radboud University Medical Center, Nijmegen, The Netherlands; Australian Primary Health Care Research Institute, Australian National University, Canberra, Australia

## Abstract

**Background:**

Most patients with advanced cancer, debilitating COPD or chronic heart failure (CHF) live at home. General practitioners (GPs) asked for guidance in how to recognize patients in need of palliative care in a timely way and to structure anticipatory care. For that reason, we developed a training for GPs in identifying patients in need of palliative care and in structuring anticipatory palliative care planning and studied its effect on out-of-hours contacts, contacts with their own GP, hospitalizations and place of death.

**Methods:**

We performed a cluster randomised controlled trial. GPs in the intervention group were trained in identifying patients in need of palliative care and anticipatory care planning. Next, for each identified patient, they were offered a coaching session with a specialist in palliative care to fine-tune a structured care plan. The GPs in the control group did not receive training or coaching, and were asked to provide care as usual.

After one year, characteristics of patients deceased of cancer, COPD or CHF in both study groups were compared with mixed effects models for out-of-hours contacts (primary outcome), contacts with their own GP, place of death and hospitalizations in the last months of their life (secondary outcomes). As a post-hoc analysis, of identified patients (of the intervention GPs) these figures were compared to all other deceased patients, who had not been identified as in need of palliative care.

**Results:**

We did not find any differences between the intervention and control group. Yet, only half of the trained GPs (28) identified patients (52), which was only 24 % of the deceased patients. Those identified patients had significantly more contacts with their own GP (B 4.5218; *p* <0.0006), were less often hospitalized (OR 0.485; p 0.0437) more often died at home (OR 2.126; p 0.0572) and less often died in the hospital (OR 0.380; p 0.0449).

**Conclusions:**

Although we did not find differences between the intervention and control group, we found in a post-hoc analysis that those patients that had been identified as in need of palliative care had more contacts with their GP, less hospitalizations, and more often died at home. We recommend future controlled studies that try to further increase identification of patients eligible for anticipatory palliative care.

The Netherlands National Trial Register: NTR2815 date 07-04-2010

## Background

In developed countries cancer, cardiovascular diseases and respiratory diseases are the main causes of death [1, 2]. Each year, in the Netherlands, about 140,000 persons die and two third of them die non-acutely of one of these three diseases (http://statline.cbs.nl/StatWeb/publication/?VW=T&DM=SLNL&PA=71594ned). Particularly in advanced stages of these diseases, symptom burden is high [3–5].

In order to improve the care for patients with incurable, life-limiting diseases, the WHO stated in 2002 that palliative care should be initiated in an early phase of the disease. A timely start facilitates anticipatory care planning in order to meet patient wishes and needs, to relieve symptoms and to prevent future symptoms and problems. Regardless of the WHO recommendation, palliative care is often restricted to a reactive approach and to the relief of physical symptoms in the terminal phase, often resulting in emergency visits by the general practitioner (GP) [6], unplanned transfers [7, 8] and hospital admissions [9, 10]. Consequently, too many patients die in another place than preferred, often with ineffective, costly and unwanted interventions [11–13].

Anticipatory care planning by earlier identification of the needs of patients in an advanced stage of their disease appeared to improve the quality of their remaining life, to decrease the number of aggressive futile interventions and depressed mood and even to prolong life in patients with advanced cancer [14–16].

Identification of patients who can benefit from anticipatory palliative care alongside or instead of disease-oriented therapies, is challenging particularly in patients with chronic obstructive pulmonary disease (COPD) or chronic heart failure (CHF), due to the fact that the course of these disease trajectories is difficult to predict [17–21]. In addition, these patients often don’t realise that they have a limited life expectancy [22]. In order to facilitate the identification of patients at risk of deterioration or dying, and thus for anticipatory palliative care, several sets of indicators have been developed, such as the Supportive and Palliative Care Indicators Tool (SPICT) in Scotland, the Prognostic Indicator Guide (PIG) in England, Neccesidades palliativas (NECPAL) in Spain and the Radboud indicators for Palliative Care needs (RADPAC) in the Netherlands [23]. These tools contain general or disease-specific indicators of decline. Examples of these indicators are repeated hospital admissions, weight loss, decrease in functional status, and the surprise question (*Would you be surprised if this patient were to die in the next twelve months?*).

In the Netherlands patients are registered with a doctor’s general practice, as part of their health care insurance. This structures a relation with a personal GP, who provides care for the large majority of their health problems presented and is the gatekeeper to specialist and hospital care. Through this structure, GPs have an overview and often intimate knowledge of the patient and his/her health conditions and social environment. This primary care-based structure plays an important role in coordinating early palliative care. GPs consider palliative care an attractive and essential part of their task, but experience difficulties with timely initiation and their coordinating role in palliative care [24, 25].

For those reasons, we developed a training for GPs in using a previously developed set of identification indicators, the RADPAC [26], in planning and providing structured anticipatory palliative care and in communicating end-of-life issues with the patient. We expected that this training would improve the care for palliative patients with cancer, COPD or CHF in the form of less contacts with the out-of-hours hours primary care cooperative, a decreased number of hospitalizations in the last three months of life, an increased number of contacts with their own GP in the last month of life and an increased number of patients that would die at home. We tested this in a cluster randomised controlled trial (RCT).

## Methods

### Design

A clustered, two-armed RCT was performed, with the GP as cluster. The study ran from February 2009 until February 2011.

### Ethical considerations

The study was approved by the research ethics committee of the Radboud University Nijmegen Medical Center in accordance with the Medical Research Involving Human Subjects Acts (WMO). It also conforms to the Helsinki Declaration.

### Participants

All GP practices in two regions of the comprehensive cancer centre of the Netherlands were invited by mail to participate. GPs were excluded if they were a consultant in a palliative care team. Participating GPs were stratified for working hours (part-time or full-time) and for degree of urbanization of their general practice (urban or rural) and they were randomly assigned to the intervention or control group by an independent statistician. To prevent contamination, GPs working together in the same practice were allocated to the same study group. Borland C software was used to randomly allocate GPs, as sequentially numbered containers, to the strata of one of both groups.

### Intervention

The intervention, described in detail elsewhere [27], consisted of three consecutive parts: 1. a five hour group training in early identification of those patients in their practice that can be considered as being palliative patients, by means of the RADPAC [26], and in proactive care planning, 2. an individual coaching session by phone with a physician specialized in palliative care, per identified palliative patient for the GP and 3. two additional peer group sessions with the GPs in the intervention group a few months after start of the intervention, with a focus on patient – GP communication regarding the initiation of a palliative care trajectory. The GPs in the intervention group were invited to use the RADPAC indicators to screen the medical records of all persons in their practice to identify those patients with CHF, COPD or cancer who potentially could benefit from a palliative care approach. They were also asked to use this screening instrument whenever new data of any patient with one of these three diseases became available, in order to timely identify the change from a curative to a palliative trajectory. They were asked to consider and start structured anticipatory palliative care for every identified patient. The GPs discussed palliative care and the study with the patient, and provided him or her a brochure with information about palliative care, the content of the study and what participation would imply for the patient.

GPs in the control group were asked to provide care as usual. Although this usual care differs per GP, they all have easy access to a large number of palliative care standards (www.Oncoline.nl), and each physician and nurse in the Netherlands can consult a specialist in palliative care 24/7 by phone [28].

### Data collection

At baseline, demographics and practice characteristics of each participating GP were collected, as well as their interest in palliative care (on a numeric rating scale (NRS) from 0, not interested at all to 10, extremely interested) and their confidence in providing palliative care by themselves (NRS from 0, not confident at all to 10, extremely confident).

If the patient was interested to participate, the GP asked him or her to sign an informed consent form, which was faxed to the researcher, together with a questionnaire with characteristics of the patient. The consulted palliative care specialists were asked to register each coaching session that they had with the GPs.

After 12 months, GPs that had not yet identified any patients for proactive palliative care were phoned by the research assistant and were asked in the form of an open question what the reason for non-inclusion was.

One year after start of the study, a questionnaire was sent to each GP in as well the intervention as the control group. They were asked to provide anonymous data, collected from the medical records of all patients that died in the past 12 months. This data included whether or not the patient died a sudden death, cause of death, age, gender, number and type of contacts with the GP out-of-hours service (by phone, consultation at the service, home visit) in the last three months before death, number and type of contacts with their own GP in the last month before death (phone calls and home visits during office hours and phone calls and home visits during out of office hours), place of death and number of hospital admissions in the last three months of life. Regarding out-of-hours contacts and contact with their own GP we chose a limited period of respectively one and three months before death, as we considered this a feasible period to check the electronic medical record, and as such contacts are most frequent near the end of life.

### Statistical analyses

The primary outcome measure was the number of contacts with the out-of-hours GP cooperative. We estimated that with 96 patients in each group, the study would have an 80 % power to detect a significance between the intervention and control group, which would be a 20 % reduction of out-of-hours contacts [27].

Statistical analysis was performed with the use of SPSS software, version 20.0 and with SAS software, version 9.2. Descriptive statistics were used to calculate frequencies, means, and standard deviations of the study variables. Differences between GPs in baseline characteristics were assessed with the use of chi-square tests for categorical variables and Student’s t-tests for continuous variables. To study differences, between deceased patients in both study groups, mixed effects models were used (SAS GLIMMIX), with the GP as a cluster. In this model, the type of disease the patient died of, the age of the GP, working hours, interest in palliative care, and estimation of the GP’s own capacity were included in the model as possible confounders.

It appeared that only a portion of the deceased patients had actually been identified as in need of palliative care in the intervention group. Therefore we performed a post-hoc analysis. We also used the same model to study differences between those patients that had been identified as in need of palliative care by GPs in the intervention group, and all other deceased patients (in as well the control and the intervention group).

## Results

**Fig. 1:**
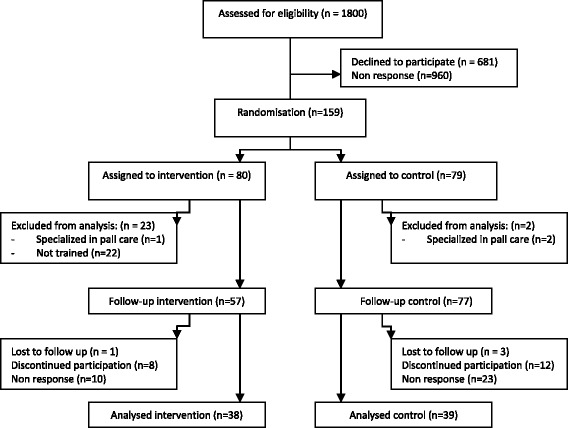
Enrolment, follow up and analyses of GPs

Within a period of one month, 159 GPs positively responded to the invitation to participate (Fig. 1). After stratification, they were randomly assigned to the intervention (*n* = 80) or control group (*n* = 79). For various reasons, 22 GPs in the intervention group were not able to join the training course. A majority of these 22 GPs worked fulltime (60 %), had slightly less years of experience (20 % had 0–5 years), worked in a group or health center (50 %), and had a larger patient list (2684 patients). Yet, the estimated number of palliative patients in this group was lower (only 23.8 % estimated to have 5 or more palliative patients per year).

Additionally, one GP from the intervention group and 2 GPs from the control group were excluded because they were already trained in specialised palliative care. For those reasons, 57 GPs received the RADPAC indicators and the training and 77 GPs were considered as control group (Fig. 1).

Of the 57 GPs that received the initial training, 28 GPs also followed the two additional peer group sessions.

The characteristics of trained GPs in the intervention and GPs in the control arm did not differ in mean age, gender, working hours, years of experience and use of a consultation service for palliative care. Also their interest in palliative care and the estimation of their own capacity to provide palliative care was the same in both groups (Table 1). Fewer GPs in the intervention arm worked in single-handed practices. The degree of urbanisation of their practice was about the same in both groups, as well as the size of their patient list.

**Table 1:** GP and practice characteristics

	Intervention	Control
	(*n* = 57)	(*n* = 77)
Age mean (sd)	48.54 (7.92)	47.85 (8.16)
Male gender n (%)	36 (63.2)	45 (58.4)
Working hours n (%)		
Fulltime	29 (50.9)	41 (53.9)
Parttime	28 (49.1)	35 (46.1)
Years experience n (%)		
0–5	6 (10.7)	10 (13.2)
6–10	11 (19.6)	10 (13.2)
≥10	39 (69.6)	56 (73.7)
Interest in pall care^1^ mean (sd)	8.21 (0.92)	8.09 (1.18)
Estimation of own capacity to provide pall care^2^ mean (sd)	6.80 (0.92)	6.85 (0.93)
Practice type n (%)		
Single-handed	9 (15.8)	19 (24.7)
Dual	28 (49.1)	26 (33.8)
Group or health center	20 (35.1)	32 (41.6)
Urbanisation degree n (%)		
High	21 (36.8)	26 (33.8)
Moderate	9 (15.8)	19 (24.7)
Low	18 (31.6)	23 (29.9)
No	9 (15.8)	9 (11.7)
Patient list mean (sd)	1710 (412)	1730 (417)
Estimated number of palliative patients/year n (%)		
≤2	4 (7.1)	7 (9.2)
3–5	29 (51.8)	43 (56.6)
5–9	20 (35.7)	23 (30.3)
≥10	3 (5.4)	3 (3.9)

^1^Interest in palliative care: numeric rating scale (NRS) from 0 (no interest at all) to 10 (extremely interested)

^2^Estimation of own capability: NRS from 0 (not capable at all) to 10 (extremely capable)

Of the 57 trained GPs in the intervention group, 28 GPs identified 52 patients (0.91 per GP; 0–4) and in 33 cases the GP had an individual coaching session with the specialist in palliative care by phone (0.58 per GP).

Twenty-nine GPs in the intervention group did not identify any suitable patient for proactive palliative care. Reasons for not having included any patients, as mentioned by the GPs during a phone call, were having had no patients that met the RADPAC indicators, difficulty initiating an appropriate discussion on proactive palliative care, or rapid deterioration or death immediately after having communicated palliative care with the patient.

Seventy-seven of the 134 GPs (57 %) returned the questionnaire with retrospective data of deceased patients: 38 of the 57 GPs (67 %) in the intervention group and 39 of the 77 GPs (48 %) in the control group. In total, data from 622 deceased patients was retrieved, of which 487 (78 %) died of cancer, COPD or CHF or a combination of these.

In the intervention (I) and control (C) group comparable numbers of patients per GP died of cancer, CHF or COPD within the past 12 months (I:5.7; C: 6.9). Mean age of the patients (I: 73; C: 74) was the same as well as the gender distribution (male patients: I: 53 %; C: 56 %).

The primary diagnosis of the deceased patients was cancer (I 70 %; C: 66 %), followed by CHF (I: 15 %; C: 19 %), COPD (I: 12 %; C: 7 %) or a combination of these diseases (I:3 %; C: 8 %) (Table 2).

**Table 2:** Patients who died of cancer, COPD or coronary heart failure (CHF) during the intervention period

	Intervention	Control
	(*N* = 216)	(*N* = 271)
Age in years (mean (sd)	73.0 (14.7)	74.0 (13.1)
Male sex n (%)	106/202 (53 %)	144/258 (56 %)
Primary diagnosis n (%)		
Cancer	152/216 (70 %)	178/271 (66 %)
CHF	32/216 (15 %)	52/271 (19 %)
COPD	26/216 (12 %)	20/271 (7 %)
Combination of cancer, COPD, CHF	6/216 (3 %)	21/271 (8 %)
Contact(s) with out-of-hours GP service last 3 months n (%)	114/209 (55 %)	157/268 (59 %)
By phone	0.79 (2.6)	0.80 (1.5)
Consultation at service	0.06 (0.3)	0.09 (0.5)
Home visits	0.85 (1.6)	1.14 (2.2)
*Total*	1.70 (3.3)	2.03 (2.9)
Contact(s) with own GP last month n (%)	188/212 (89 %)	250/268 (93 %)
By phone office hours	2.33 (2.9)	2.71 (3.0)
Home visits office hours	5.59 (5.1)	4.42 (3.6)
By phone out of office hours	0.19 (0.7)	0.10 (0.5)
Home visits out of office hours	0.46 (1.1)	0.38 (1.1)
*Total*	8.58 (7.5)	7.62 (6.0)
Location of death n (%)		
Home	103/210 (49 %)	124/267 (46 %)
Hospital	67/210 (32 %)	78/267 (29 %)
Nursing home	13/210 (6 %)	16/267 (6 %)
Care home	12/210 (6 %)	21/267 (8 %)
Hospice	14/210 (7 %)	25/267 (9 %)
Other location	1/210 (1 %)	3/267 (1 %)
Hospital admission(s) last 3 months n (%)	116/210 (55 %)	159/255 (62 %)
Hospital admissions last 3 months mean (sd)	0.82 (0.9)	0.89 (0.9)

Data are mean (sd) or n/N (%). Some percentages do not sum to 100 % because of rounding

In the mixed model analyses, we found intra cluster coefficients (ICCs) from 0.04 (dying at home) to 0.14 (out-of-hours contacts). We found no differences between the intervention and control group in the number of contacts with the GP out-of-hours cooperative in that last three months, nor in the number of contacts a patient had with their own GP in the last month, hospitalisations in the last three months, dying at home or dying in the hospital (Table 3).

**Table 3:** Mixed effects model estimating the effect of intervention

		B^1^	95 % CI	p
*Number of contacts with out-of-hours GP service last 3 months*				
	Intervention group	0.4828	−0.733 − 1.698	0.4307
	Cause of death^2^			0.4599
	CHF	−0.1016	−0.806 – 0.603	
	COPD	−0.4729	−1.403 – 0.457	
	Combination	0.6775	−0.515 – 1.870	
	GP works fulltime	1.0229	−0.181 – 2.227	0.0946
	Age of GP^3^	−0.0792	−0.155 − −0.003	0.0418
	Interest in palliative care^3^	−0.8286	−1.461 − −0.196	0.0110
	Self-efficacy regarding PC^3^	0.3191	−0.384 − 1.022	0.3679
*Number of contacts with own GP last month*				
	Intervention group	0.7155	−0.935 − 2.366	0.3901
	Cause of death^2^			<.0001
	CHF	−3.8582	−5.434 − −2.283	
	COPD	−5.0800	−7.173 − −2.987	
	Combination	−4.1761	−6.883 − −1.470	
	GP works fulltime	−1.1778	−2.803 – 0.447	0.1526
	Age of GP^3^	−0.0035	−0.108 – 0.101	0.9474
	Interest in palliative care^3^	0.3770	−0.477 – 1.231	0.3813
	Self-efficacy regarding PC^3^	−0.6855	−1.649 – 0.278	0.1602
	Intervention group			
		OR^4^	95 % CI	p
*Hospitalisation(s) last three months yes/no*				
	Intervention group	0.797	0.464 – 1.372	0.4078
	Cause of death^2^			0.0132
	CHF	0.729	0.423 – 1.256	
	COPD	2.300	1.056 – 5.011	
	Combination	3.165	1.092 – 9.172	
	GP works fulltime	0.917	0.539 – 1.561	0.7467
	Age of GP^3^	1.017	0.983 – 1.053	0.3207
	Interest in palliative care^3^	0.802	0.606 – 1.061	0.1202
	Self-efficacy regarding PC^3^	1.227	0.895 – 1.682	0.1993
*Dying at home yes/no*				
	Intervention group	1.130	0.646 − 1.976	0.6655
	Cause of death^2^			<.0001
	CHF	0.393	0.227 – 0.682	
	COPD	0.174	0.075 – 0.405	
	Combination	0.167	0.059 – 0.472	
	GP works fulltime	1.359	0.784 – 2.356	0.2697
	Age of GP^3^	0.995	0.960 – 1.030	0.7644
	Interest in palliative care^3^	1.109	0.833 – 1.477	0.4721
	Self-efficacy regarding PC^3^	0.776	0.558 – 1.079	0.1295
*Dying in hospital yes/no*				
	Intervention group	1.095	0.684 – 1.754	0.7012
	Cause of death^2^			0.0009
	CHF	1.501	0.862 – 2.611	
	COPD	3.692	1.870 – 7.292	
	Combination	2.617	1.102 – 6.217	
	GP works fulltime	0.863	0.541 – 1.376	0.5303
	Age of GP^3^	0.991	0.962 – 1.021	0.5468
	Interest in palliative care^3^	1.021	0.800 – 1.303	0.8654
	Self-efficacy regarding PC^3^	1.254	0.936 – 1.680	0.1271

1) B = difference of means

2) cause of death (cancer, CHF, COPD or combination of those; cancer is reference group), age of GP, working hours, interest in palliative care, and estimation of own capacity were included in the model as possible confounders

3) Effects of continuous variables are assessed as one unit offsets from the mean

4) OR = odds ratio

There was a relation found between the cause of death and the number of contacts that the patient had with their own GP within the last month of life (*p* < 0.0001) (Table 3). For patients with cancer, the number of contacts in the last month of life with their own GP was higher than for patients with CHF, COPD or a combination of these diseases. Cause of death was also related to having had at least one hospitalization in the last three months of life (p 0.0132). Patients with COPD were more often hospitalized, as well as those patients with a combination of these diseases, while patients with CHF had smaller odds to be hospitalized. Finally, the cause of death was related to dying at home (*p* < 0.0001), and to dying in the hospital (p 0.0009). Patients with cancer died more often at home and less often in the hospital than patients with one of the other causes of death.

### Post-hoc analysis: identified patients versus all other patients

Of the 52 patients that had been identified as in need of palliative care, there were 50 with available data; two patients had moved and it was not possible to follow up on their status. One patient (with COPD) was still alive at the moment that the retrospective data was collected. Data of the remaining 49 deceased patients was compared to data of all other patients from GPs in the intervention as well as the control group (*n* = 437) that died of cancer, COPD or CHF or a combination of these.

Table 4 shows that the identified patients were younger than the other patients (64.8 versus 74.6 years). Also their primary diagnosis differed; the identified patients were more likely to have cancer (86 versus 66 %), and less often CHF (4 % versus 19 %).

**Table 4:** Difference between those patients that were identified as in need of palliative care and all others

	Deceased patients who were identified as in need of palliative care	Deceased patients without those identified
	(*N* = 49)	(*N* = 428)
Age mean (sd)	64.8 (12.1)	74.6 (13.7)
Male sex n (%)	30/49 (61 %)	219/409 (54 %)
Primary diagnosis n (%)		
Cancer	42/49 (86 %)	287/436 (66 %)
CHF	2/49 (4 %)	82/436 (19 %)
COPD	4/49 (8 %)	41/436 (9 %)
Combination of cancer, COPD, CHF	1/49 (2 %)	26/436 (6 %)
Contact(s) with out-of-hours GP service last 3 months n (%)	24/47 (51 %)	247/430 (57 %)
By phone	1.26 (4.9)	0.74 (1.4)
Consultation at service	0.04 (0.2)	0.08 (0.4)
Home visits	0.64 (1.1)	1.05 (2.1)
*Total*	*1.94 (5.2)*	*1.88 (2.8)*
Contact(s) with own GP last month n (%)	45/49 (92 %)	393/431 (91 %)
By phone office hours means	3.14 (3.8)	2.47 (2.8)
Home visits office hours means	8.27 (6.7)	4.55 (3.8)
By phone out of office hours	0.46 (1.2)	0.11 (0.5)
Home visits out of office hours	1.02 (1.7)	0.35 (1.0)
*Total mean*	*13.00 (9.8)*	*7.48 (6.0)*
Location of death n (%)		
Home	33/49 (67 %)	194/428 (45 %)
Hospital	7/49 (14 %)	138/428 (32 %)
Nursing home	5/49 (10 %)	24/428 (6 %)
Care home	0/49 (0 %)	33/428 (8 %)
Hospice	4/49 (8 %)	35/428 (8 %)
Other location	0/49 (0 %)	4/428 (1 %)
Hospital admission(s) last 3 months n (%)	20/48 (42 %)	255/417 (61 %)
Hospital admissions last 3 months mean (sd)	0.60 (1.0)	0.89 (0.9)

Data are mean (sd) or n/N (%). Some percentages do not sum to 100 % because of rounding

The number of contacts in the last three months of life with the out-of-hours GP service was the same in both groups. Yet, the identified patients had more contacts with their own GP in the last month of life (13.00 versus 7.48). Also the location of death differed: the identified patients died at home more often (67 versus 45 %) and less often in the hospital (14 versus 32 %) (Table 4).

Finally, a smaller percentage of the identified patients had had at least one hospitalisation in the last three months of their life as compared to the other patients (42 versus 61). The mean number of hospital admissions of identified patients was also lower (0.60 versus 0.89).

We found no differences in number of contacts with the out-of-hours GP cooperative in the clustered model, controlled for GP clusters, cause of death (cancer, CHF, COPD or a combination of these), the GP working part-time or fulltime, age of the GP, interest in palliative care and self-efficacy regarding providing palliative care (Table 5). However, there was a difference in the number of contacts that identified patients had with their own GP in the last month before death (p 0.0006). They were less often hospitalized in the last three months of life (p 0.0437), and died less often in the hospital (p 0.0449). Although they also died more often at home, this difference was not statistically significant (p 0.0572).

**Table 5:** Mixed effects model estimating the effect of being identified as in need of palliative care

		B^1^	95 % CI	p
*Number of contacts with out-of-hours GP cooperative last 3 months*				
	Identified patients	−0.0832	−1.156 – 0.989	0.8695
	Cause of death^2^			0.4831
	CHF	−0.1240	−1.833 – 0.585	
	COPD	−0.470	−1.404 – 0.464	
	Combination	0.6421	−0.553 – 1.837	
	GP works fulltime	1.0132	−0.164 – 2.190	0.0904
	Age of GP^3^	−0.0776	−0.152 − −0.003	0.0410
	Interest in palliative care^3^	−0.8049	−1.422 − −0.188	0.0113
	Self-efficacy regarding PC^3^	0.3545	−0.328 – 1.037	0.3033
*Number of contacts with own GP last month*				
	Identified patients	4.5218	2.336 – 6.707	0.0006
	Cause of death^2^			<0.0001
	CHF	−3.5003	−5.054 − −1.947	
	COPD	−4.8675	−6.920 − −2.815	
	Combination	−3.9826	−6.633 − −1.332	
	GP works fulltime	−1.3109	−2.865 – 0.243	0.0970
	Age of GP^3^	−0.0008	−1.101 – 0.099	0.9876
	Interest in palliative care^3^	0.4259	−0.387 – 1.238	0.2993
	Self-efficacy regarding PC^3^	−0.8122	−1.730 – 0.105	0.0817
	Intervention group			
		OR^4^	95 % CI	p
*Hospitalisation(s) last three months yes/no*				
	Identified patients	0.485	0.215 – 0.975	0.0437
	Cause of death^2^			0.0117
	CHF	0.682	0.395 – 1.179	
	COPD	2.227	1.017 – 4.879	
	Combination	3.105	1.074 – 8.973	
	GP works fulltime	0.939	0.557 – 1.583	0.8094
	Age of GP^3^	1.018	0.984 – 1.054	0.2937
	Interest in palliative care^3^	0.792	0.603 – 1.041	0.0935
	Self-efficacy regarding PC^3^	1.241	0.912 – 1.688	0.1663
*Dying at home yes/no*				
	Intervention group	2.126	0.974 – 4.643	0.0572
	Cause of death^2^			<0.0001
	CHF	0.418	0.240 – 0.726	
	COPD	0.177	0.076 – 0.414	
	Combination	0.172	0.061 – 0.485	
	GP works fulltime	1.321	0.762 – 2.291	0.3166
	Age of GP^3^	0.995	0.960 – 1.030	0.7629
	Interest in palliative care^3^	1.116	0.839 – 1.483	0.4465
	Self-efficacy regarding PC^3^	0.761	0.549 – 1.055	0.1000
*Dying in hospital yes/no*				
	Identified patients	0.380	0.148 – 0.975	0.0449
	Cause of death^2^			0.0011
	CHF	1.361	0.781 – 2.371	
	COPD	3.867	1.928 – 7.757	
	Combination	2.423	1.025 – 5.726	
	GP works fulltime	0.884	0.554 – 1.409	0.5991
	Age of GP^3^	0.989	0.960 – 1.019	0.4619
	Interest in palliative care^3^	1.031	0.809 – 1.313	0.8025
	Self-efficacy regarding PC^3^	1.309	0.980 – 1.748	0.0678

1) B = difference of means

2) cause of death (cancer, CHF, COPD or combination of those; cancer is reference group), age of GP, working hours, interest in palliative care, and estimation of own capacity were included in the model as possible confounders

3) Effects of continuous variables are assessed as one unit offsets from the mean

4) OR = odds ratio

## Discussion

This study reports the results of an RCT on the effects of training GPs in early identification of patients that could profit from palliative care and in structured anticipatory palliative care planning. There was no difference found in the primary outcome: the number of consultations with the out-of-hours cooperative was the same for the intervention and control group. Also no effects were found in the secondary outcome parameters: other types of contact with their own GP, hospital admissions and place of death. Yet, in the intervention group only 24 % of the patients that died of cancer, CHF or COPD, had been identified as in need of palliative care. In addition, only half of the trained GPs actually identified patients, and coaching sessions were requested for only a part of patients. For those reasons, we performed a post-hoc analysis in which we studied the outcomes regarding those patients that had actually been identified by the trained GPs as in need of palliative care in comparison to all the other patients from the intervention and control group. The identified patients had had more contact with their own GP in the last month of life, had half the chance to have a hospitalisation in the last three months of life, had less than half the chance to die in hospital, and twice the chance of dying at home, although the latter was not statistically significant. Of the identified patients 67 % died at home. In a recent study concerning place of death of home-dwelling patients that died after a protracted terminal illness, 52.5 % of the Dutch patients died at home, which is a much lower occurrence than of our identified patients [29].

The mean age of the identified patients was ten years younger than that of the other patients. This may have influenced the place of death, as younger patients are more likely to have a partner who can take care of them which may have increased their chance of dying at home. Also in an English study, the chance of dying at home or in a hospice was higher for younger patients [30]. Yet, in a German study, younger patients had a higher chance to die in hospital [31]. The influence of age on the outcome is therefore unclear.

The positive effects of the intervention regarding those patients that had had anticipatory palliative care is in accordance with several other RCTs. In a Canadian nurse-led study, early palliative care for patients with advanced cancer had a significant effect on parameters such as quality of life and depressed mood [15]. However, they did not find an effect on the number of hospitalizations. In an American study, Temel et al. found that early palliative care resulted in an improved quality of life and mood, less aggressive interventions and prolonged survival in patients with advanced lung cancer [14]. In a cluster-RCT in Canada, Zimmermann et al. found a significant improvement in quality of life at the end of life and satisfaction with care, but no significant effect on the quality of life as measured with the Facit-spiritual well being of advanced cancer patients 3 months after inclusion [16]. All three studies were restricted to patients with cancer, and in all three studies patients were included via hospitals, in contrast to our study. In these studies, the intervention was hospital-based and delivered directly to the patients. In our study GPs were included as participants, not patients. We provided them with training and tools to identify and proactively plan the care for palliative patients.

Yet, only half of the trained GPs in the intervention group identified patients in need of palliative care, and the identified number per GP was only a fraction of the number of patients that died per GP during this period. These aspects indicate an identification problem with regard to the RADPAC tool and/or an inclusion threshold. Secondly, and probably related to the limited number of identified patients: our intervention addressed GPs, while it is often a medical specialist who remains the primary caregiver for patients in their final stage of life. This is related to the fact that patients still receive disease-oriented interventions. In such cases, training GPs without actively involving the medical specialists will hardly influence the identification of palliative patients or palliative care planning. Finally, although GPs are the experts in discussing end-of-life aspects with their patients, and our intervention further strengthened this expertise, the reality of daily life still is, that timely palliative care and involving the patient in anticipating a care trajectory remains difficult, particularly in non-cancer patients. Seeing as the entire disease trajectory of patients with COPD can span 15–20 years, and of patients with CHF spans 5–10 years, which is much longer than the trajectory of most patients with cancer, changing such a long-term relation will require more efforts than just a training on anticipatory palliative care. Janssen et al. described in 2011 that for several reasons, the quality of communication about end of life issues with patients with COPD needs to be improved [22]. Also in our study, more patients with cancer had been included than patients with COPD or CHF. This was also one of the conclusions of a review on palliative patient-GP communication [32].

### Strengths and weaknesses

This is the first RCT to study the effect of training GPs in identifying patients in need of palliative care and in providing structured palliative care planning. We were able to include many GPs in a very short period of time, which confirms their interest in palliative care [24, 33].

Although we planned to perform an intention-to-treat analysis, the GPs that were allocated to the intervention group but did not follow the training, were lost to follow up. The characteristics of these GPs differed slightly; they were more likely to work fulltime and they had a larger patient list. Being too busy probably caused their absence. Next, only 57 % of the GPs returned the questionnaire with patient data, and therefore we could not include their deceased patients in the analyses. Thirdly, the positive effects we found regarding those patients that had been identified as in need of palliative care, were found in a post-hoc analysis, which has not been described in the study protocol. These results need to be explored further in future studies, as we don’t know whether these differences are caused by the intervention or because the identified patients are a selected group. Lastly, it was not possible to prospectively register and monitor patient data in the control group, and thus of having quality of life as outcome measure.

## Conclusions

This study shows that the design we chose was not completely compatible with an intervention that combined identification of patients in need of palliative care and anticipatory care planning. Only a portion of the eligible patients had been identified, and in this subgroup a post-hoc analysis showed positive effects of having identified patients as in need of palliative care and providing anticipatory palliative care. For that reason, the absence of differences between the entire intervention and control group does not show a failure of anticipatory palliative care; it reflects a low number of identified patients.

We have used the insights from this study to adapt our methodology and to ensure that patients are timely identified as in need of palliative care. These adaptations are currently being applied in a prospective study on proactive supportive care in patients with COPD. Patients are recruited by the attending specialist at the moment of hospitalization for an acute exacerbation COPD, palliative care is provided by a dedicated hospital palliative care team, and the primary outcome is quality of life [34].

## Abbreviations

CHF: coronary heart failure
COPD: chronic obstructive pulmonary disease
GP: general practitioner
NECPAL: Neccesidades palliativas
PIG: Prognostic Indicator Guide
RADPAC: Radboud Indicators for Palliative Care Needs
RCT: randomised controlled trial
SPICT: Supportive and Palliative Care Indicators Tool
WHO: World Health Organisation

## References

[1] van der Velden LF, Francke AL, Hingstman L, Willems DL (2009). Dying from cancer or other chronic diseases in the Netherlands: ten-year trends derived from death certificate data. BMC Palliat Care.

[2] Franks PJ, Salisbury C, Bosanquet N, Wilkinson EK, Lorentzon M, Kite S, Naysmith A, Higginson IJ (2000). The level of need for palliative care: a systematic review of the literature. Palliat Med.

[3] Philip J, Lowe A, Gold M, Brand C, Miller B, Douglass J, Sundararajan V (2012). Palliative care for patients with chronic obstructive pulmonary disease: exploring the landscape. Intern Med J.

[4] Teunissen SC, Wesker W, Kruitwagen C, de Haes HC, Voest EE, de Graeff A (2007). Symptom prevalence in patients with incurable cancer: a systematic review. J Pain Symptom Manage.

[5] Pantilat SZ, O’Riordan DL, Dibble SL, Landefeld CS (2012). Longitudinal assessment of symptom severity among hospitalized elders diagnosed with cancer, heart failure, and chronic obstructive pulmonary disease. J Hosp Med.

[6] Worth A, Boyd K, Kendall M, Heaney D, Macleod U, Cormie P, Hockley J, Murray S (2006). Out-of-hours palliative care: a qualitative study of cancer patients, carers and professionals. Br J Gen Pract.

[7] van den Block L, Deschepper R, Drieskens K, Bauwens S, Bilsen J, Bossuyt N, Deliens L (2007). Hospitalisations at the end of life: using a sentinel surveillance network to study hospital use and associated patient, disease and healthcare factors. BMC Health Serv Res.

[8] Burge FI, Lawson B, Critchley P, Maxwell D (2005). Transitions in care during the end of life: changes experienced following enrolment in a comprehensive palliative care program. BMC Palliat Care.

[9] Klinkenberg M, Visser G, van Groenou MI, van der Wal G, Deeg DJ, Willems DL (2005). The last 3 months of life: care, transitions and the place of death of older people. Health Soc Care Community.

[10] van den Block L, Deschepper R, Bilsen J, van Casteren V, Deliens L (2007). Transitions between care settings at the end of life in belgium. JAMA.

[11] Meeussen K, Van den Block L, Bossuyt N, Bilsen J, Echteld M, Van Casteren V, Deliens L (2009). GPs’ awareness of patients’ preference for place of death. Br J Gen Pract.

[12] Abarshi E, Onwuteaka-Philipsen B, Donker G, Echteld M, Van den BL, Deliens L (2009). General practitioner awareness of preferred place of death and correlates of dying in a preferred place: a nationwide mortality follow-back study in the Netherlands. J Pain Symptom Manage.

[13] Lynn J, Teno JM, Phillips RS, Wu AW, Desbiens N, Harrold J, Claessens MT, Wenger N, Kreling B, Connors AF (1997). Perceptions by family members of the dying experience of older and seriously ill patients. SUPPORT Investigators. Study to Understand Prognoses and Preferences for Outcomes and Risks of Treatments. Ann Intern Med.

[14] Temel JS, Greer JA, Muzikansky A, Gallagher ER, Admane S, Jackson VA, Dahlin CM, Blinderman CD, Jacobsen J, Pirl WF, Billings JA, Lynch TJ (2010). Early palliative care for patients with metastatic non-small-cell lung cancer. N Engl J Med.

[15] Bakitas M, Lyons KD, Hegel MT, Balan S, Brokaw FC, Seville J, Hull JG, Li Z, Tosteson TD, Byock IR, Ahles TA (2009). Effects of a palliative care intervention on clinical outcomes in patients with advanced cancer: the Project ENABLE II randomized controlled trial. JAMA.

[16] Zimmermann C, Swami N, Krzyzanowska M, Hannon B, Leighl N, Oza A, Moore M, Rydall A, Rodin G, Tannock I, Donner A, Lo CI (2014). Early palliative care for patients with advanced cancer: a cluster-randomised controlled trial. Lancet.

[17] Hanratty B, Hibbert D, Mair F, May C, Ward C, Capewell S, Litva A, Corcoran G (2002). Doctors’ perceptions of palliative care for heart failure: focus group study. BMJ.

[18] Goodlin SJ, Hauptman PJ, Arnold R, Grady K, Hershberger RE, Kutner J, Masoudi F, Spertus J, Dracup K, Cleary JF, Medak R, Crispell K, Piña I, Stuart B, Whitney C, Rector T, Teno J, Renlund DG (2004). Consensus statement: Palliative and supportive care in advanced heart failure. J Card Fail.

[19] O’Leary N, Murphy NF, O’Loughlin C, Tiernan E, McDonald K (2009). A comparative study of the palliative care needs of heart failure and cancer patients. Eur J Heart Fail.

[20] Jaarsma T, Beattie JM, Ryder M, Rutten FH, McDonagh T, Mohacsi P, Murray SA, Grodzicki T, Bergh I, Metra M, Ekman I, Angermann C, Leventhal M, Pitsis A, Anker SD, Gavazzi A, Ponikowski P, Dickstein K, Delacretaz E, Blue L, Strasser F (2009). Palliative care in heart failure: a position statement from the palliative care workshop of the Heart Failure Association of the European Society of Cardiology. Eur J Heart Fail.

[21] Curtis JR (2008). Palliative and end-of-life care for patients with severe COPD. Eur Resp J.

[22] Janssen DJ, Curtis JR, Au DH, Spruit MA, Downey L, Schols JM, Wouters EF, Engelberg RA (2011). Patient-clinician communication about end-of-life care for Dutch and US patients with COPD. Eur Respir J.

[23] Maas EA, Murray SA, Engels Y, Campbell C (2013). What tools are available to identify patients with palliative care needs in primary care: a systematic literature review and survey of European practice. BMJ Support Palliat Care.

[24] Groot MM, Vernooij-Dassen MJ, Crul BJ, Grol RP (2005). General practitioners (GPs) and palliative care: perceived tasks and barriers in daily practice. Palliat Med.

[25] Meijler WJ, Van Heest F, Otter R, Sleijfer DT (2005). Educational needs of general practitioners in palliative care: outcome of a focus group study. J Cancer Educ.

[26] Thoonsen B, Engels Y, van Rijswijk E, Verhagen S, van Weel C, Groot M, Vissers K (2012). Early identification of palliative care patients in general practice: development of RADboud indicators for PAlliative Care Needs (RADPAC). Br J Gen Pract.

[27] Thoonsen B, Groot M, Engels Y, Prins J, Verhagen S, Galesloot C, van Weel C, Vissers K (2011). Early identification of and proactive palliative care for patients in general practice, incentive and methods of a randomized controlled trial. BMC Fam Pract.

[28] Groot MM, Vernooij-Dassen MJ, Courtens AM, Kuin A, van der Linden BA, van Zuylen L, Crul BJ, Grol RP (2005). Requests from professional care providers for consultation with palliative care consultation teams. Support Care Cancer.

[29] De Roo ML, Miccinesi G, Onwuteaka-Philipsen BD, Van Den Noortgate N, Van den Block L, Bonacchi A, Donker GA, Lozano Alonso JE, Moreels S, Deliens L, Francke AL (2014). Actual and preferred place of death of home-dwelling patients in four European countries: making sense of quality indicators. PLoS One.

[30] Gao W, Ho YK, Verne J, Glickman M, Higginson IJ, project GUC (2013). Changing patterns in place of cancer death in England: a population-based study. PLoS Med.

[31] Simon ST, Gomes B, Koeskeroglu P, Higginson IJ, Bausewein C (2012). Population, mortality and place of death in Germany (1950–2050) - implications for end-of-life care in the future. Public Health.

[32] Slort W, Blankenstein AH, Schweitzer BP, Knol DL, Deliens L, Aaronson NK, van der Horst HE (2013). Effectiveness of the ACA (Availability, Current issues and Anticipation) training programme on GP-patient communication in palliative care; a controlled trial. BMC Fam Pract.

[33] Meijler WJ, Van Heest F, Otter R, Sleijfer DT (2005). Educational needs of general practitioners in palliative care: outcome of a focus group study. J Cancer Educ.

[34] Duenk RG, Heijdra Y, Verhagen SC, Dekhuijzen RP, Vissers KC, Engels Y (2014). PROLONG: a cluster controlled trial to examine identification of patients with COPD with poor prognosis and implementation of proactive palliative care. BMC Pulm Med.

